# Meeting abstracts from the 2022 ANZAED conference

**DOI:** 10.1186/s40337-024-00993-2

**Published:** 2024-03-20

## O1. The cognitive behavioural therapy scale for eating disorders (CBTS-ED): a tool for clinicians, supervisors, and researchers to evaluate and improve patient outcomes

### Bronwyn Raykos^1^, Tracey D. Wade^2^, Zafra Cooper^7^, Philip Masson^3^, Victoria A. Mountford^4^, Rebecca Murphy^5^, Madeleine Tatham^3^, Jennifer J. Thomas^6^, Hannah M. Turner^8^, and Glenn Waller^3^

#### ^1^Centre for Clinical Interventions, Northbridge, WA, Australia; ^2^Flinders University, Bedford Park, SA, Australia; ^3^Sheffield University, Sheffield, United Kingdom; ^4^Kings College, St Lucia QLD, Australia, London, United Kingdom; ^5^Oxford, Sydney NSW, Oxford, United Kingdom; ^6^Harvard University, Cambridge, Massachusetts, United States; ^7^Yale, New Haven, CT, United States; ^8^Southern Health NHS, Hampshire, United Kingdom

##### **Correspondence:** Bronwyn Raykos (bronwyn.raykos@health.wa.gov.au); Tracey D. Wade (tracey.wade@flinders.edu.au)

*Journal of Eating Disorder* 2024, **12(1):** O1

**Background:** Current efforts by NEDC and ANZAED to implement a credentialing system for eating disorder clinicians aims to achieve a national minimum standard for training in evidence-based interventions. However, there is substantial evidence that clinicians do not reliably deliver core components of evidence-based interventions for eating disorders, even if they have received training, without ongoing supervision.

**Objective:** The present paper describes the development of a fidelity scale, the Cognitive Behavioural Therapy Scale for Eating Disorders (CBTS-ED), that aims to measure core competences for delivery of cognitive behavioural therapy for eating disorders (CBT-ED), aligning with national and international standards, that can be used in supervision.

**Method:** A group of 10 international experts in CBT-ED, collaborated to identify key competences for delivery of CBT-ED and develop a valid and reliable scale with high clinical utility to assess competence in CBT-ED.

**Results:** The present paper will describe the core principles of the CBTS-ED; and initial data examining reliability of expert and non-expert ratings of therapy sessions rated using the CBTS-ED scale.

**Discussion:** The potential utility of the CBTS-ED scale as a tool for self-reflection, training and supervision as well as evaluation of competence and adherence to CBT-ED, and ultimately improving patient outcomes will be discussed.

## O2. Disordered eating prevention: co-designing an education session for use in community child health services

### Lyza Norton^1^, Joy Parkinson^1^, Neil Harris^1^, and Laura Hart^2^

#### ^1^Griffith University, Southport, QLD, Australia; ^2^University of Melbourne, Parkville, VIC, Australia

##### **Correspondence:** Lyza Norton (lyza.norton@griffithuni.edu.au)

*Journal of Eating Disorder* 2024, **12(1):** O2

Currently limited information is available to parents about “how” to communicate with young children about food to promote healthful eating behaviours. This study aimed to co-design an education session for parents of children under 2 years of age to decrease the likelihood of child risk factors for disordered eating. Previous research highlights the conversations parents have during mealtimes can influence children’s eating habits into the future.

This qualitative study was based on co-design principles and the Knowledge to Action Framework. After co-design, the education sessions were implemented with parents at the Child Health Services, to evaluate program feasibility and acceptability.

First, this study engaged with Child Health Nurses (CHNs) (n = 15) to develop a group education session for parents of infants about how to develop healthy eating habits. Resources with evidenced-based strategies for parents were co-designed by the research team and CNHs.

Second, an uncontrolled repeated measures pilot trial was conducted to examine the feasibility and acceptability of the resources for parents (n = 50) and CHNs. Parent knowledge was measured via pre and post online questionnaires. The acceptability of the education session and accompanying resources was via online questionnaires and semi-structured interviews with a subset of parents. The intervention included an education session delivered with PPT slides and group discussions, with accompanying resources (feeding bib and quick resource guide). Findings from parents indicated high acceptability of and engagement with the program. This study contributes to our understanding of “how” to co-design preventive health resources with health professionals.

## O3. Understanding what parental factors predict the use of coercive feeding practices

### Lyza Norton^1^, Joy Parkinson^1^, Neil Harris^1^, and Laura Hart^2^

#### ^1^Griffith University, Southport, QLD, Australia; ^2^University of Melbourne, Parkville, VIC, Australia

##### **Correspondence:** Lyza Norton (lyza.norton@griffithuni.edu.au)

*Journal of Eating Disorder* 2024, **12(1):** O3

Early feeding experiences influence future eating habits for children. Understanding which parental factors predict engagement in particular feeding practices is central for designing preventive interventions. Early feeding education has traditionally focused on the “what” and “when” of nutrition, but more information on “how” parents can feed their children healthfully may help children develop positive relationships with food. Our study examined the relationship between parents’ use of coercive food parenting practices (pressure to eat, restriction) and levels of disordered eating, food literacy, Body Mass Index (BMI) and socio-economic status (SES) among mothers. Mothers living in Australia, with young children completed an online questionnaire (n = 819). A series of multiple linear regression analysis was conducted between the two dependent variables (pressure to eat and restriction) and the predictor variables (food literacy, disordered eating, BMI and SES).

Results showed maternal eating disorder symptoms were the strongest predictor of pressure to eat feeding practices. There was a significant negative relationship between pressure to eat and both food literacy and BMI. Both low-income categories also had a significant positive relationship to pressure to eat.

Examining restriction there was a significant positive relationship with eating disorder symptoms and a significant negative relationship with BMI.

Results suggest current early feeding education, with its focus on improving nutrition literacy, is inadequate. Future programs need to focus on reducing maternal disordered eating, and coaching parents in “how” to talk about food and to effectively promote the development of positive eating patterns in children.

## O4. Increasing the reach: Family Inclusive Treatment for Adults Affected by Eating Disorders

### Carmel Fleming PhD^1^

#### ^1^Senior Social Worker, Clinical Educator, Superivsor and Family Worker, Queensland Eating Disorder Service and Clinical Lecturer, School of Nursing, Midwifery and Social Work, University of Queensland, Brisbane, Australia.

##### **Correspondence:** Carmel Fleming PhD (carmel.fleming@health.qld.gov.au)

*Journal of Eating Disorder* 2024, **12(1):** O4

Reports of family or other supports of adults with eating disorders being routinely included in treatment are rare despite patients highlighting the importance of psychosocial support during recovery and carers reporting problems accessing and interacting with services. This reveals a gap in the field regarding factors that may promote family inclusive practice with adults. This presentation describes a multiphase mixed method study into the involvement of family of origin or family of choice of adults attending a typical outpatient service; the feasibility and effect of a brief family intervention on target problems; and acceptability when offered in addition to treatment-as-usual. Results discussed include patient and carer quality of life, burden, and psychosocial concerns as well experiences of the intervention. The findings reveal that adult carers remain available to support treatment despite experiencing considerable burden and distress and that adults are willing to involve carers and keen to participate in relational models of care if supported to do so. In addition, the rate of carer involvement was twice as high when family inclusion was actively promoted and when family consultations was offered as part of routine clinical delivery than when ad hoc carer contact occurred at the service. The planned, structured nature of the family work intervention, the supportive neutral environment, and the collaborative, solution-focused process were highly valued by participants. This indicates the field may benefit from broader recognition of the relevance of relational approaches in adult treatment. Suggestions for more responsive service delivery in this area will be discussed.

## O5. Butterfly body bright: pilot evaluation of a whole-school body image program for Australian primary schools

### Stephanie R Damiano^1,2^, Hannah K Jarman^2,3^, Danni Rowlands^1^, and Susan J Paxton^2^

#### ^1^Butterfly Foundation, Crows Nest, NSW, Australia; ^2^La Trobe University, Sydney, NSW, Australia; ^3^Deakin University, Geelong, VIC, Australia

##### **Correspondence:** Stephanie R Damiano (stephanie.damiano@butterfly.org.au)

*Journal of Eating Disorder* 2024, **12(1):** O5

Childhood is a crucial period for the development of body attitudes and behaviours. Schools have an important role in influencing key risk and protective factors for children’s body image. A pilot evaluation was conducted on Australia’s first whole of primary school body image program, Butterfly Body Bright; designed to promote positive body image and healthy attitudes and behaviours towards the body, eating, and physical activity in an appearance-inclusive environment. The aim of the evaluation was to assess the impact of some of the teacher-led Body Bright lessons on the body image and attitudes of 114 Year 4–6 students (50.4% male, 48.8% female, 0.8% other) from two campuses of an independent school in Melbourne, Victoria. Twelve classes were randomly assigned to receive one Body Bright lesson (BRAVE, RESILIENT, or GRATEFUL). Students completed pre-lesson, post-lesson, and 3–7-week follow-up surveys that measured their state and trait body image and related attitudes. Pre-post analyses showed significant improvement in student’s state body image, body appreciation, confidence to deal with appearance-teasing, and intention to seek help if they were having a hard time. Students who received the RESILIENT lesson showed a significant improvement in media literacy. Students who received the BRAVE lesson showed significant improvements in intention to avoid appearance comparisons and in trait body image at follow-up. Body Bright lessons show potential to improve children’s body image and related attitudes, but further evaluation is required. Results are encouraging for the potential impact of Butterfly Body Bright when the whole program is adopted.

## O6. Oral Ketamine and Zinc Supplementation Trial for Anorexia Nervosa

### Rosiel Elwyn^1^, Jules Mitchell^1^, Adem T. Can^1^, Megan Dutton^1^, Jim Lagopoulos^1^, Daniel Hermens^1^

#### ^1^University of the Sunshine Coast, Sippy Downs, QLD, Australia; Thompson Institute, Birtinya QLD, Australia

##### **Correspondence:** Rosiel Elwyn (Rosiel.Elwyn@research.usc.edu.au); Jules Mitchell (Jules.Mitchell@research.usc.edu.au)

*Journal of Eating Disorder* 2024, **12(1):** O6

This study is an open-label, dose-ranging clinical trial aiming to explore the safety, feasibility and tolerability of a sub-anaesthetic dose of oral ketamine and combined zinc supplementation for individuals with low and higher weight anorexia nervous (AN and ‘atypical’ AN) of all gender identities aged 18-30 years. Participants will undergo 6 weeks of a combined novel psycho-pharmaceutical treatment, with continual supplementation for the remainder 6 weeks of the trial to support neuroplastic brain changes (synaptic remodelling). Medical, mental health and cognitive assessments and brain imaging will occur at study entry and at two additional time points. Gut microbiome fecal sampling collection will occur at study entry and at 6 weeks of the combined treatment. The primary objective is to determine the safety, feasibility and tolerability of the novel psycho-pharmaceutical treatment in young participants diagnosed with early onset, chronic AN. Additionally, it aims to determine the influence of the treatment on low and higher weight AN in psychological, cognitive and behavioural domains, on interoceptive function and on the gut microbiome. Exploratory objectives include gut microbial profiles, neuroimaging and blood biomarker correlates associated with and/or predictive of treatment response.

## O7. Creative solutions to overwhelming demand

### Dr Tania Withington^1^, Claire Mihalopoulos^2^, Victoria Brown^1^, Michelle Boyd^1^, and Dr Salvatore Catania^1^

#### ^1^Children's Health Queensland, South Brisbane, QLD, Australia; Child and Youth Mental Health Service, Hornsby, NSW, Australia; Eating Disorder Program, Phillip, ACT, Australia; ^2^Children's Health Queensland, Service Improvement, South Brisbane, QLD, Australia

##### **Correspondence:** Tania Withington (EatingDisorders@health.qld.gov.au)

*Journal of Eating Disorder* 2024, **12(1):** O7

Between July 2019 and June 2021, Children's Health Queensland has experienced a surge in children and young people presenting with eating disorders and disordered eating across the hospital and health service. New referrals have increased between 40 and 150 percent across community and inpatient services, often with higher rates of complexity at first presentation. In response to this demand, several strategies have been employed to identify service impacts, increase service capacity, build capacity in local community mental health teams and innovate waitlist management strategies. Acknowledging the vital role consumers have, a consumer carer coordination role has been established to enhance the services offered to patients and families and participate in service leadership. The presentation will outline the whole of health service clinical redesign process, and the concurrent innovations developed to respond to demand pressures across the continuum of care from inpatient services to specialist community eating disorders services.

## O8. The functions of binge eating in binge eating disorder: development of an eight-factor scale and its associations with childhood maltreatment

### Elyse O'Loghlen^1^, Roslyn Galligan^1^, and Sharon Grant^1^

#### ^1^Swinburne University of Technology, Parramatta, NSW, Australia

##### **Correspondence:** Elyse O'Loghlen (elyseologhlen@swin.edu.au)

*Journal of Eating Disorder* 2024, **12(1):** O8

Within binge eating disorder (BED), binge eating is widely understood as a response to dietary restraint and/or emotion dysregulation. However, qualitative literature suggests that additional functions of binge eating exist within this population. The present study developed the Functions of Binge Eating Scale (FBES) to quantitatively validate these reported functions of binge eating in BED. 3sw3sw3sw3sw3sw3swand childhood maltreatment, considering the significant proportion of the BED population with a trauma history. An initial item pool developed by the authors was rated by Australian researchers and healthcare professionals within the eating disorder field (n = 22). The refined item pool was administered to adults with self-reported binge eating (n = 882), alongside related measures of validity (e.g., Eating Beliefs Questionnaire-18). Results of exploratory and confirmatory factor analyses supported an eight-factor scale (emotion regulation, hedonic hunger, dietary restraint, numbness/dissociation, emotion expression, self-punishment, control, self-protection). The scale demonstrated good internal reliability and adequate construct validity. As hypothesised, associations were found between types of childhood maltreatment and functions (e.g., sexual abuse and self-protection). These results extend the currently limited research literature regarding the wide-ranging functions of binge eating within BED. These findings may also alert clinicians to explore and target more complex processes that perpetuate this behaviour, including the potential use of interventions outside of gold standard BED treatments (e.g., CBT-E) as clinically indicated by the presenting function; for instance, the use of trauma-focussed therapy for trauma-related functions.

## O9. My face feels like yours: exploring enfacement in females with varying levels of eating disorder symptomatology during COVID-19

### Jade Portingale^1^, David Butler^2^, Jessica Chadakorn^2^, Jan Teh^1^, Milly Tupthim^2^, Lili Vincent^1^, Zhen An^1^, Litza Kiropoulos^1^ & Isabel Krug^1^

#### ^1^School of Psychological Sciences, Melbourne, VIC, Australia; The University of Melbourne, Parkville, VIC, Australia; ^2^Cairnmillar Institute, Hawthorn East, VIC, Australia

##### **Correspondence:** Jade Portingale (jade.portingale@unimelb.edu.au)

*Journal of Eating Disorder* 2024, **12(1):** O9

Individuals with eating disorder (ED) symptomatology typically hold disturbances in body perception, which may be attributable to broader impairments in multisensory integration. Indeed, ED populations demonstrate greater susceptibility to embodiment illusions (i.e., they are more likely to incorporate another person’s body part into their own self-image). Curiously, experiencing embodiment illusions with healthy (i.e., not underweight) models has led to improvements in body satisfaction and self-perception. Despite one’s face representing a highly salient and distinctive physical feature, it remains unknown whether individuals with ED symptomatology will show the above patterns in relation to enfacement illusions (i.e., embodiment involving one’s face). The current study investigated whether individuals with high ED risk, relative to low ED risk, show greater susceptibility to enfacement, and whether enfacement improves self-perception among both groups. Using a novel online procedure, female participants completed self-perception measures before and after an enfacement task which involved mimicking (or not mimicking) facial expressions of a healthy-weight model. Whilst data collection is ongoing, preliminary analyses of 104 participants revealed that somewhat inconsistent with past research, objective enfacement occurred irrespective of the synchrony of stimulation. Contrary to predictions, subjective enfacement was greater following synchronous (relative to asynchronous) mimicry, ED risk groups did not differ in their susceptibility to enfacement, and no improvements in self-perception emerged post-intervention. Nonetheless, this is the first study to our knowledge to demonstrate that facial mimicry effects self-perception. Our online enfacement measures hold implications for ED research and clinical practice, particularly within contexts where face-to-face contact is restricted (i.e., COVID-19).

## O10. Embodiment as a function of, and method to improve, eating disorder symptomatology in clinical and non-clinical samples: a systematic review

### Jade Portingale^1^, Isabel Krug^1^, Hermione Liu^1^, Jessica Chadakorn^2^, Milly Tupthim^2^, Kayla Purnomo^1^, Litza Kiropoulos^1^, and David Butler^2^

#### ^1^School of Psychological Sciences, Melbourne, VIC, Australia; The University of Melbourne, Parkville, VIC, Australia; ^2^Cairnmillar Institute, Hawthorn East, VIC, Australia

##### **Correspondence:** Jade Portingale (jade.portingale@unimelb.edu.au)

*Journal of Eating Disorder* 2024, **12(1):** O10

Individuals with eating disorder (ED) symptomatology tend to hold disturbances in body perception. Growing research evidence suggests that such disturbances may be attributable to broader impairments in multisensory integration, as made evident by their increased susceptibility to embodiment illusions (i.e., incorporating another person’s body or body part into one’s own self-representation). Research also suggests that experiencing embodiment with healthy-weight models can temporarily improve body perception in individuals with ED symptomatology by manipulating the processes underlying self-perception. However, despite growing interest, this is still an emerging area of research. No published research to date has systematically reviewed current findings related to embodiment procedures to elucidate whether individuals experiencing ED symptomatology exhibit specific deficits and/or distortions in self-perception, and whether embodiment illusions can improve self-perception in individuals with ED symptomatology. These were the central aims of the current review. A systematic search of six international databases—PsycINFO, MEDLINE, EMBASE, Web of Science, CINAHL, and ProQuest—identified 29 studies that met inclusion criteria. Among these, 13 addressed the first aim, and 22 addressed the second aim. The findings provide support for greater susceptibility to embodiment illusions among those with ED symptomatology, and the potential for these illusions to improve body perception among clinical and sub-clinical ED populations. Given the limitations surrounding current ED interventions, these findings hold important implications for the development of alternative treatment approaches for ED patients, and prevention and early intervention strategies targeting those at risk of developing an ED.

## O11. SupportED eProgram evaluation: skills-based online self-help program for carers of people with an eating disorder

### Dr. Jane Miskovic-Wheatley^1^, Jasmin Schlage^2^, Rachel Simeone^1^, Melissa Pehlivan^1^, Prof Caroline Hunt^2^, and A/Prof. Sarah Maguire^1^

#### ^1^InsideOut Institute for Eating, The University of Sydney, Camperdown, NSW, Australia; ^2^School of Psychology, The University of Sydney, Camperdown, NSW, Australia

##### **Correspondence:** Jane Miskovic-Wheatley (insideout.research@sydney.edu.au)

*Journal of Eating Disorder* 2024, **12(1):** O11

Carers’ inclusion within treatment for people with a lived experience of eating disorders (ED) is a well-recognised gap, with carers often experiencing distress, burden and uncertainty in their caregiving knowledge and skills. The SupportED eProgram is an online skill-based program for carers that provides information about EDs, communication, recovery facilitation, emotion and burnout management skills. The objective of this study was to evaluate the effectiveness, feasibility, and acceptability of SupportED through program engagement and outcomes including carers’ levels of depression, anxiety, stress, caregiving burden, knowledge, skills, confidence, and willingness to care for someone with an ED. Outcome data and program feedback were collected pre- and post-program and at three-month follow-up. Preliminary analysis indicates that following program completion, carers demonstrated significant reductions in guilt and dysregulated behaviour, significant improvements in caregiving knowledge, skills, confidence, and willingness, and significant improvements in module-specific knowledge and skills. Carers found the program content and online format acceptable and useful, however, suggested improvements include information on diverse presentations, realistic examples, time efficiency, and ease of navigation/access. Results suggest the SupportED eProgram is acceptable, effective, and feasible for supporting carer knowledge, skill development, and reducing aspects of carer burden. These findings emphasise the role of online programs in supporting carers of someone with an ED and provide insight into further tailoring programs to the carer needs.

## O12. “You don't fall through the cracks”: a thematic analysis of patient perspectives of team-based outpatient eating disorder treatment

### Megan Bray^1^, Gabriella Heruc^2^, and Olivia Wright^1^

#### ^1^School of Human Movement and Nutrition Sciences, The University of Queensland, St Lucia, QLD, Australia; ^2^Eating disorders and Nutrition Research Group (ENRG) and School of Medicine, Western Sydney University, Penrith, NSW, Australia

##### **Correspondence:** Megan Bray (m.bray@uq.edu.au)

*Journal of Eating Disorder* 2024, **12(1):** O12

Clinical practice guidelines for eating disorders recommend collaborative team-based treatment. However, such approaches are inconsistently implemented in practice and patient preferences have not been explored. This study aimed to collate perspectives of team-based eating disorder treatment among eating disorder patients to inform clinical practice. Semi-structured interviews were conducted with 27 individuals who had received team-based eating disorder treatment from a mental health professional, dietitian, and general practitioner in the past two years. Diagnostic categories of participants included anorexia nervosa, bulimia nervosa, other specific feeding and eating disorder, and binge eating disorder. Interview data were inductively analysed using thematic analysis. Participants shared that: (i) quality team-based care was difficult to access unless referred via one health professional to another with whom they had an established relationship; (ii) the general practitioner, dietitian and mental health professional were essential members of the treatment team; (iii) team cohesion accelerated positive treatment outcomes; (iv) treatment outcomes improved when health professionals had complementary roles; (v) team-based care enhanced engagement of patients’ “healthy self” in treatment. Overall, it was agreed that cohesive, complementary team-based eating disorder treatment from a general practitioner, dietitian and mental health professional improved patient engagement and treatment outcomes. Given patients’ desire for services of this nature, development and empirical testing of clearly defined team-based eating disorder treatment models is warranted.

## O13. Towards interprofessional collaborative practice: a thematic analysis of clinician perspectives of team-based outpatient eating disorder treatment

### Megan Bray^1^, Gabriella Heruc^2^, and Olivia Wright^1^

#### ^1^School of Human Movement and Nutrition Sciences, The University of Queensland, St Lucia, QLD, Australia; ^2^Eating disorders and Nutrition Research (ENRG) and School of Medicine, Western Sydney University, Penrith, NSW, Australia

##### **Correspondence:** Megan Bray (m.bray@uq.edu.au)

*Journal of Eating Disorder* 2024, **12(1):** O13

Team-based treatment from a general practitioner, mental health professional and dietitian is recommended within clinical practice guidelines. Despite these recommendations, the clinician perspective of team-based care, which ranges from siloed multidisciplinary treatment (MDT) to synergetic Interprofessional Collaborative Practice (ICP), has not been explored. This study aimed to collate perspectives on team-based treatment from eating disorder clinicians to inform collaborative practice. Semi-structured interviews were conducted with 18 eating disorder clinicians with greater than two years’ experience providing team-based outpatient eating disorder treatment. Professional backgrounds of participants included dietitians (n = 9), mental health professionals (n = 6) and general practitioners (n = 3). Interview questions were guided by the core competencies for ICP: values/ethics; roles/responsibilities; interprofessional communication; and teamwork. Data were inductively analysed using thematic analysis. Participants shared that: (i) ICP improved quality of care compared to MDT; (ii) ICP decreased burden of care for clinicians; (iii) frequent and informal communication promoted ICP; (iv) lack of remuneration for communication was a barrier to ICP; (v) Lack of interprofessional education was a barrier to establishing and sustaining ICP. ICP, rather than MDT, may be an important strategy to improve eating disorder treatment, although financial and educational barriers to its implementation remain. Given the desire for interprofessional collaborative care from eating disorder clinicians, its incorporation into treatment approaches is warranted, alongside empirical testing.

## O14. How do we support the uptake of evidence-based treatment for eating disorders: listening to clinicians about the key barriers and enablers

### Dr Emma Spiel^1^, Dr Beth Shelton^1^, Dr Sarah Trobe^1^, Dr Gabriella Heruc^2^, and Dr Siân McLean^2,3^

#### ^1^National Eating Disorders Collaboration, Crows Nest, NSW, Australia; ^2^Australia and New Zealand Academy for Eating Disorders, St Leonards, NSW, Australia; ^3^La Trobe University, Sydney, NSW, Australia

##### **Correspondence:** Dr Emma Spiel (training@nedc.com.au); Dr Sarah Trobe (training@nedc.com.au)

*Journal of Eating Disorder* 2024, **12(1):** O14

Comprehensive guidelines and standards which describe best practice care, including the use of empirically supported treatment approaches for eating disorders exist within the Australian context (i.e., ANZAED 2020, NEDC, 2018). Nonetheless, availability of consensus guidelines does not guarantee their uptake into practice, a process strongly influenced by contextual factors, not just intervention effectiveness. To improve access to evidence-based treatment and care for people experiencing eating disorders in Australia, more needs to be known about the individual and contextual barriers and enablers to implementation of evidence-based care after skill-development initiatives such as training and supervision. This research aims to address this gap.

**Methods:** Survey data assessing individual and organisational barriers and enablers to implementation of evidence-based treatment was collected from n = 500) mental health (n = 307) and dietetic (n = 173) clinicians receiving a professional development package of training and supervision as part of the ANZAED Eating Disorder Credential project. Analyses identified the most strongly endorsed barriers and enablers to implementation.

**Results:** Clinicians were asked to indicate from a list whether a described factor was either (1 = not a barrier, 2 = Minor barrier, 3 = Moderate barrier or 4 = Major barrier. The most frequently endorsed major barriers to implementing evidence-based treatment across the group were funding (23%) and clinician lack of knowledge (22%). Clinicians working within regional/rural/remote settings more frequently endorsed systemic major barriers to implementing an eating disorders treatment response than those working in metro settings; those in public settings endorsed greater systemic barriers than those in private, and clinicians in general mental health reported greater systemic barriers than those in eating disorder specific services. Workplace culture and attitudinal factors were the most frequently endorsed major systemic enablers for implementing an eating disorders treatment response, followed by knowledge of eating disorders. Clinicians working within Eating Disorder Specific Services identified the presence of substantially more enablers than those in general health or mental health services, as did those in private practice compared with clinicians working in public settings.

**Conclusions:** Findings suggest that appropriate service funding and clinician skill development initiatives will likely address some of the key barriers to implementing and sustaining a whole of system approach to eating disorders care. Organisations should pay careful attention to building culture and leadership to enable clinicians to utilize their skills and sustain an evidence-informed service response.

## O15. A snapshot of the current eating disorders workforce in Australia: Who is ready, willing, and able? Characteristics of clinicians upskilling in eating disorders treatment

### Dr Emma Spiel^1^, Dr Beth Shelton^1^, Dr Sarah Trobe^1^, Dr Gabriella Heruc^2^, and Dr Siân McLean^2,3^

#### ^1^National Eating Disorders Collaboration, Crows Nest, NSW, Australia; ^2^Australia and New Zealand Academy for Eating Disorders, St Leonards, NSW, Australia; ^3^La Trobe University, Sydney, NSW, Australia

##### **Correspondence:** Dr Emma Spiel (training@nedc.com.au); Dr Sarah Trobe (training@nedc.com.au)

*Journal of Eating Disorder* 2024, **12(1):** O15

Developing an effective, accessible system of care for people experiencing eating disorders requires coordination and intervention at multiple levels. Workforce development is a key intervention and initiatives must be tailored to the needs of consumers, the clinician, the service context, and to enhancing capability to deliver safe and effective treatment and care for people experiencing eating disorders. This research will aim to determine the characteristics of the Australian mental health and dietetic eating disorders workforce associated with perceived skill, confidence, and willingness to provide treatment for people experiencing eating disorders.

**Methods:** Participants were n = 480 clinicians (307 mental health clinicians and 173 dietitians) who completed a funded professional development package of training and supervision as part of the ANZAED Eating Disorder Credential system. Survey data was collected prior to participants receiving their professional development package assessing demographic characteristics and perceived competency and attitudes towards the provision of eating disorder treatment.

**Results:** Clinician demographic data showed that fewer clinicians provided services for ARFID (40%) compared to Binge Eating Disorder (62%), Anorexia Nervosa (62%) and Bulimia Nervosa (58%). Majority of clinicians provided services for cisgender women and girls (85%), as well as gay, lesbian, and bisexual individuals (79%), cisgender boys and men (85%), neurodivergent individuals (74%), and trans or non-binary individuals (62%), with fewer providing treatment for Aboriginal and Torres Strait Islander people (51%). Aside from those working in ED specific services, mental health clinicians and dietitians typically coordinated care across service settings. Mental health clinicians (95%) were also much more likely to receive supervision than dietitians (73%). On a 5-point scale (1 = very limited knowledge, 5 = excellent knowledge) most clinicians rated their skill to provide eating disorder treatment as either 2 = limited (24%) 3 = average (31%) or 4 = good (25%). Dietitians rated their perceived knowledge, skill, and willingness to provide treatment for eating disorders higher than did mental health clinicians, with group differences reaching statistical significance. Most clinicians reported being either 4 = willing (29%) or 5 = very willing (50%) to provide treatment.

**Conclusions:** Clinician willingness to provide treatment appears to be a key lever to attracting clinicians to the workforce through skill development initiatives. Broader research into the demographics and needs of the workforce is needed to inform data-driven workforce development initiatives.

## O16. Exploring the experience of receiving dietetic care for eating disorders in primary care: perspectives of consumers and carers

### Alana Heafala^1,2^, Lana J. Mitchell^1,2^ and Lauren Ball^1,2^

#### ^1^School of Health Sciences and Social Work, Gold Coast Campus, Griffith University, Southport, QLD, Australia; ^2^Menzies Health Institute Queensland, Gold Coast Campus, Griffith University, Southport, QLD, Australia

##### **Correspondence:** Alana Heafala (alana.heafala@griffithuni.edu.au)

*Journal of Eating Disorder* 2024, **12(1):** O16

**Background:** Dietitians are important members of eating disorder (ED) treatment teams. Previous research indicates little is currently known about the experience of receiving nutrition care for EDs. This study aimed to explore aspects of dietetic care perceived as important by consumers and carers seeking treatment for EDs.

**Method:** Semi-structured interviews were used to explore the experiences and perceptions of individuals aged ≥ 15 years, who (i) had lived experience of EDs or (ii) provided care for someone with an ED and had received care from a primary care dietitian. Inductive thematic analysis was used to analyse data from interview transcripts. Synthesised member checking assessed whether participants felt the identified themes and subthemes sufficiently reflected their experiences and perceptions.

**Results:** Twenty-four consumers (n = 21) and carers (n = 3) participated. Three key themes and twelve subthemes were identified from the data: (1) valuing a person-centred approach to dietetic care; (2) the therapeutic alliance is central to engaging in dietetic care; and (3) sharing the complex journey. Member checking was completed by 17 participants, and all supported the identified themes and subthemes.

**Discussion:** This study advances the understanding of what consumers and carers perceive as the most important aspects of dietetic care for EDs. Person-centredness, empathy, trust and collaboration within ED care were valued and facilitated ongoing engagement with dietitians. The findings can be used by dietitians and other health professionals to inform practice and further understanding of what positively influences the therapeutic alliance in ED treatment.

## O17. Co-occurring disordered eating and substance use: A systematic review of prevalence and associations with trauma

### Ivana Kihas^1^, Emma L. Barrett^1^, Stephen W. Touyz^2,3^, Maree Teesson^1^, and Katherine L. Mills^1^

#### ^1^The Matilda Centre for Research in Mental Health and Substance Use, The University of Sydney, Sydney, NSW, Australia; ^2^School of Psychology, The University of Sydney, Sydney, NSW, Australia; ^3^Inside Out Institute, The University of Sydney, Sydney, NSW, Australia

##### **Correspondence:** Ivana Kihas (ivana.kihas@sydney.edu.au)

*Journal of Eating Disorder* 2024, **12(1):** O17

**Background:** Eating disorder (ED), substance use disorder (SUD) and post-traumatic stress disorder typically have their onset in adolescence. Research has shown that these disorders frequently co-occur and suggests that trauma exposure may be one shared risk factor that increases their vulnerability of developing. Substances are often used to suppress appetite and self-medicate negative affective mood states. Likewise, disordered eating patterns have been linked to the development and maintenance of substance-related problems. We conducted a systematic review to explore the relationship patterns, prevalence and temporal sequencing of co-occurring substance use and disordered eating among young people and the degree to which the role of trauma exposure has been examined in relation to these comorbidities.

**Methods:** A systematic review of electronic databases Ovid MEDLINE, PsycINFO, Embase, CINAHL, ProQuest, Informit, Scopus, Web of Science, PubMed and Cochrane Library; from 2008 to 2018.

**Results:** 5546 records were identified through database searching, 1975 duplicates were removed. 3571 titles and abstracts were screened. 513 full-text studies were assessed for eligibility. Data was extracted from 63 studies. Data extraction included methodology, prevalence, patterns of the comorbidity, the underlying relationships and whether impacts of trauma were assessed.

**Conclusion:** This is the first systematic review to assess the prevalence and patterns of comorbid substance use and disordered eating, assessing whether the impact of trauma among young people was measured. Better understanding of this comorbidity can impact treatment provisions and outcomes; encouraging more inclusive treatment options and more comprehensive prevention strategies for young people at risk of developing these comorbid disorders.

## O18. Study protocol and preliminary findings for an outcome evaluation of residential treatment for eating disorders

### Sinead Day^1^, Rebekah Rankin^1^, Phillipa Hay^1^, Janet Conti^2^, Lucie Ramjan^3^, Kathy Tannous^1,4^, Deborah Mitchison^1^, Warren Ward^5,6^, Katherine Gill^7^, Cathy Mihalopoulos^8^, Long Le^8^, Haider Mannan^1^, Aunty Kerrie Doyle^9^, Liz Dale^10^, and Kirsten McMahon^6^

#### ^1^Translational Health Research Institute, School of Medicine, Western Sydney University, Penrith, NSW, Australia; ^2^School of Psychology, Western Sydney University, Penrith, NSW, Australia; ^3^School of Nursing and Midwifery, Western Sydney University, Penrith, NSW, Australia; ^4^School of Business, Western Sydney University, Penrith, NSW, Australia; ^5^University of Queensland, St Lucia, QLD, Australia; ^6^Queensland Eating Disorder Service (QuEDS), Royal Brisbane and Women's Hospital, Brisbane, QLD, Australia; ^7^Foundations for Success, City East, QLD, Australia; ^8^Division of Health Economics, School of Public Health and Preventive Medicine, Monash University, Clayton, VIC, Australia; ^9^School of Medicine, Western Sydney University, Penrith, NSW, Australia; ^10^University of Wollongong, Wollongong, NSW, Australia

##### **Correspondence:** Sinead Day (s.day2@westernsydney.edu.au); Rebekah Rankin (rebekahmjrankin@gmail.com)

*Journal of Eating Disorder* 2024, **12(1):** O18

Residential treatment facilities offer a new treatment setting within the continuum of care for individuals with eating disorders but are limited by a lack of robust research evidence. This presentation will outline the study protocol for a clinical evaluation of the Wandi Nerida residential care facility—the first residential service for the treatment of eating disorders in Australia. The evaluation will employ a mixed-method between groups design with multiple aims: (1) to investigate the effectiveness of this residential program in reducing eating disorder and comorbid psychopathology and improving quality of life and functioning; (2) to explore the experiences of individuals undergoing treatment at Wandi Nerida, as well as those of their carers and service providers; and (3) determine the cost-effectiveness of this treatment approach. Quantitative and qualitative data will be collected concurrently from 2021 to 2024. Preliminary demographic and descriptive data are available for the current sample of 31 individuals aged 18-40 years (M = 26.9 years, SD = 6.0). Most participants to date report an eating disorder diagnosis of Anorexia Nervosa (93.3%). Current data indicate lower self-reported eating disorder psychopathology at post-treatment compared to pre-treatment, as reflected in the Eating Disorder Examination Questionnaire (EDE-Q) total score (M = 4.86 [SD 1.57] pre-treatment, M = 3.64 [SD 1.17] post-treatment) and frequency of eating disorder behaviours (e.g., for purging, M = 4.77 [SD 7.82] pre-treatment, M = 2.08 [SD 4.76] post-treatment). Further preliminary data will be presented along with details of the study protocol.

## O19. Does #beauty have a dark side and can self-compassion protect against body dysmorphic concerns and cosmetic surgery consideration?

### Dr Veya Seekis^1^, Grace Barker^1^

#### ^1^Griffith University, Nathan, QLD, Australia

##### **Correspondence:** Veya Seekis (v.seekis@griffith.edu.au); Grace Barker (grace.barker@griffithuni.edu.au)

*Journal of Eating Disorder* 2024, **12(1):** O19

The disconcerting rise of body dysmorphic concerns and increasing rates of cosmetic procedures in young women may be due to societal pressure placed on the importance of female appearance. In line with sociocultural theories, ample research shows that one influential mode of societal pressure on women’s appearance is social media. However, extant research is yet to examine the growing trend of beauty content on social media and its association with body dysmorphic concerns and cosmetic surgery consideration. Given that women who experience a preoccupation with perceived appearance flaws may also develop an eating disorder in trying to alter their appearance, it is important to examine protective factors such as self-compassion. A sample of 401 undergraduate women aged 17–25 years (Mage = 19.36) completed measures of beauty social media engagement, upward appearance comparison, general attractiveness internalisation, body dysmorphic concerns, cosmetic surgery consideration, and self-compassion. In line with the tripartite influence model, path analysis supported a serial mediation model that comprised significant paths from beauty social media engagement through in turn, upward appearance comparison, general attractiveness internalisation, and body dysmorphic concerns, to cosmetic surgery consideration. Moderated mediation analyses revealed that self-kindness buffered the indirect link between beauty social media and both body dysmorphic concerns and cosmetic surgery consideration via upward appearance comparison. Findings provide new insights into (a) the underlying links between beauty social media, body dysmorphic concerns, and cosmetic surgery consideration and (b) how self-kindness may provide a shield against these outcomes when engaging in comparison processes.

## O20. Eating disorders day program in the time of COVID-19’: staff and consumer perspectives of a telehealth model of service delivery

### Emma Gallagher^1^, Brigette Lupton^1^

#### ^1^Eating Disorders Day Program, James Fletcher Hospital, Newcastle NSW

##### **Correspondence:** Emma Gallagher (HNELHD-EDDayProg@health.nsw.gov.au); Brigette Lupton (HNELHD-EDDayProg@health.nsw.gov.au)

*Journal of Eating Disorder* 2024, **12(1):** O20

The Eating Disorders Day Program (EDDP) has historically operated in a face-to-face semi-closed group format. The program is delivered to up to eight clients at a time over a period of up to 12 weeks. Consumers receive meal support for the provided breakfasts, morning teas, and lunches, as well as CBT-E and DBT group therapy. The COVID-19 lockdowns in 2020–21 however, prompted the EDDP to shift to a telehealth model of service delivery.

The EDDP hypothesised there was clinical and logistical potential in a telehealth model for clients however this would not suit all consumers. A quality improvement project was undertaken to examine staff and consumers’ experiences of the telehealth model of EDDP.

A mixed-method case study approach was used for this project. Data was collected using qualitative interviews, consumer feedback surveys, and quantitative outcome measures. Qualitative data was collated, coded and analysed thematically to identify themes, while quantitative data was used to reflect clinical change.

Findings from this project highlight consumer and staff perceptions of the strengths, weaknesses, opportunities and threats of the telehealth model compared to standard face-to-face service delivery. Themes around collaborative weighing inconsistency, the limits of enforcing treatment non-negotiables remotely, managing engagement while recognising screen fatigue, balancing increased client control with reduced food variety exposure and the benefits of generalising skills to the home environment are all explored.

Learnings from these case studies may be used to inform future day program treatment planning and implementation, particularly in instances where face-to-face service is not feasible.

## O21. Goodform: outcomes of a pilot trial of a boys’ body image and supplement prevention program with 14–16 year-old boys in Australia

### A/Prof. Zali Yager^1,2^, Dr. Jo R Doley^1^, Dr. Siân McLean^3^, and Dr. Scott Griffiths^4^

#### ^1^Institute for Health and Sport, Victoria University, Melbourne, VIC, Australia; ^2^Body Confident Collective, Boambee, NSW, Australia; ^3^La Trobe University, Melbourne, VIC, Australia; ^4^University of Melbourne, Melbourne, VIC, Australia

##### **Correspondence:** A/Prof. Zali Yager (zali@bodyconfidentcollective.org)

*Journal of Eating Disorder* 2024, **12(1):** O21

After 30 years of body image and eating disorder research, we now have body image programs that are efficacious for girls. However, few programs have shown consistently positive findings for boys, and programs delivered in co-educational settings have not generally improved outcomes for all genders.

Our aim in this study was to evaluate Goodform, a 4-week, teacher delivered program that we developed specifically for boys in order to improve their body image and reduce intentions to use muscle-building supplements. The program was based on cognitive dissonance, social learning theory, and a social norms approach, and drawn from two body image programs that have shown promise for males (The Body Project- More than Muscles, and the Athletes Training and Learning to Avoid Steroids Program, ATLAS).

Participants were N = 596 boys, in grade 9 and 10 at secondary schools in Australia participated in the study. Schools were randomly assigned to the intervention or wait-list control group and students completed online questionnaires at one-week pre-test, immediate post, and 3-month follow up. Analyses involving multi-level, mixed-effect regression models revealed no changes over time attributable to the intervention, with the exception of increased reporting regarding pressure to be thin in the intervention condition.

Our results have implications for clinicians and researchers in the body image and eating disorder prevention fields. Lessons learned in this study can inform future school-based efforts for reducing muscle building supplement use in terms of the content and methods adopted in this work.

## O22. Wandi Nerida: a residential treatment for people with eating disorders—the facility, the model, the program

### Dr Catherine Houlihan^1^, Carly Roukos^1^, Dr Ranjani Utpala^2^

#### ^1^Wandi Nerdia, Mooloolah Valley, QLD, Australia; ^2^The Butterfly Foundation, Crows Nest, NSW, Australia

##### **Correspondence:** Carly Roukos (Carly.Roukos@wandinerida.org.au); Ranjani Utpala (ranjani@lotusblue.au)

*Journal of Eating Disorder* 2024, **12(1):** O22

Wandi Nerida opened in July 2019 as Australia’s first residential treatment service for people with eating disorders. Owned and operated by the Butterfly Foundation, Wandi Nerida means “gather together to blossom” and implements the Butterfly Foundation Residential Eating Disorders Treatment (B-FREEDT) Model of Care^©^ (MoC). This presentation will describe how the B-FREEDT MoC, the treatment program, and the physical environment provide holistic support to its participants to support our overall mission—to help make recovery a reality.

Set on 25 acres of beautiful bushland in the Sunshine Coast hinterland, Wandi Nerida provides a 7-day per week, breakfast-to-bedtime program including evidence-based and best practice interventions such as cognitive-behavioural therapy, nutrition education, and process-driven groups as well as expressive and complimentary therapies such as art therapy and equine-assisted mental health. This presentation will explore the initial experiences of Wandi Nerida’s first year of operating and key learnings from both our participants and multi-disciplinary team.

Wandi Nerida is currently being evaluated through a robust clinical and economic evaluation conducted by Western Sydney University (WSU). This presentation will complement one by the team at WSU who will present on preliminary findings from Wandi Nerida’s first year.

## O23. Making sense of eating disorders continues: screening for sensory modulation differences

### Dr. Elysa Roberts^1^, Sunee Hedges^1^, and Dr. Alison E. Lane^1,2^

#### ^1^Occupational Therapy Program, University of Newcastle, College of Health, Medicine and Wellbeing, School of Health Sciences, Newcastle, NSW, Australia; ^2^Olga Tennison Autism Research Centre, La Trobe University, College of Science, Health, Engineering, School of Psychology & Public Health, Melbourne, VIC, Australia

##### **Correspondence:** Dr. Elysa Roberts (elysa.roberts@newcastle.edu.au)

*Journal of Eating Disorder* 2024, **12(1):** O23

Sensory modulation, i.e., ability to regulate the intensity of response to sensory stimuli, is an emerging neurobiological factor in the expression of symptoms of eating disorders. Currently, there is no validated measure of sensory modulation variability for individuals with symptoms of an eating disorder. The Glasgow Sensory Questionnaire (GSQ) is a psychometrically sound assessment of the frequency of sensory modulation differences, i.e., hyper- or hypo-reactivity, among adults in the general population and/or presenting with traits of autism spectrum disorder. Therefore, this study assessed the content validity of the GSQ within a population of adults with symptoms of eating disorders. Adults (mostly female) participants (n = 690) were allocated to asymptomatic (n = 346) or symptomatic (n = 344) groups based on at-risk scores on the EAT-26. Content validity was assessed using exploratory factor analysis on the GSQ scores of the symptomatic group. One factor contributed to 22.58% of the variance and was supported by 28 loading items. Results did not reveal a specific pattern of sensory modulation differences based on sensory domain or modality. Secondary analysis of the 28-item subset found a significant difference (p < .000) between asymptomatic (M = 34.74; SD = 14.4) and symptomatic groups (M = 46.98; SD = 17.2). Thus, it appears a single factor consisting of a reduced 28-item subset best represents the construct of sensory modulation for this population and can discriminate between individuals with and without eating disorder symptoms. Emerging constructs within the reduced item set and implications of having a validated instrument to screen if an individual is experiencing sensory modulation differences will be discussed.

## O24. Hidden in plain sight: health presentation patterns of people with eating disorders to an acute medical hospital

### Susan Hart^1,2^, Devlin Higgins^1^, Lauren Christie^1,3^

#### ^1^St Vincent's Health Network, Sydney, NSW, Australia; ^2^Eating Disorders & Nutrition Research Group, Eating Disorders and Body Image, Western Sydney University, Penrith, NSW, Australia; ^3^Faculty of Health Sciences, Australian Catholic University, Fitzroy, VIC, Australia

##### **Correspondence:** Susan Hart (susan.hart@svha.org.au)

*Journal of Eating Disorder* 2024, **12(1):** O24

**Aim:** There are limited descriptions of clinical and demographic data on people with eating disorders (EDs) when presenting to acute medical hospitals. Patients may be reluctant to disclose an ED and present with unspecified mental health or physical complaints such as low weight, syncope, or abdominal pain. A clearer picture of presentation patterns is needed to address the unmet need of people with EDs who are presenting to generalist health services.

**Methods:** Medical record audits were undertaken for confirmed or suspected presentations of EDs to a large metropolitan hospital between 2018 and 2020. Diagnostic codes used to retrieve data included ‘eating disorder unspecified’, hypokalaemia or bradycardia. Identified cases relied on documentation in medical records of an ED diagnosis, description of ED behaviors, laboratory abnormalities i.e., unexplained hypokalaemia or other features characteristic of an ED.

**Results:** 258 individuals were identified with confirmed or suspected ED over 3 years who had a total of 447 emergency and 453 inpatient presentations. Most inpatient presentations were in medical (n = 203) and mental health wards (n = 172) with few cases admitted primarily for ED treatment (n = 24) or its side effects (n = 38).

**Discussion:** There are greater numbers of people presenting with EDs than is expected when looking at current health utilisation data. A minority of cases are diagnosed, and few receive treatment targeting the ED. More training and education are needed to identify signs and symptoms of EDs, in addition to awareness of the true burden of disease in acute medical hospitals to improve clinical outcomes for people with EDs.

## O25. Barriers to the delivery of evidence-based care for eating disorders at an acute medical hospital

### Susan Hart^1,2^, Sophie Smith^3^, Devlin Higgins^1^, and Lauren Christie^1,4^

#### ^1^St Vincent's Health Network Sydney, Darlinghurst, NSW, Australia; ^2^Eating Disorders & Nutrition Research Group, Eating Disorders and Body Image, Western Sydney University, Penrith, NSW, Australia; ^3^Flinders University, Bedford Park, SA, Australia; ^4^Faculty of Health Sciences, Australian Catholic University, Thebarton, SA, Australia

##### **Correspondence:** Susan Hart (susan.hart@svha.org.au)

*Journal of Eating Disorder* 2024, **12(1):** O25

**Aim:** There are gaps in training, knowledge and confidence when managing people with eating disorders (EDs) in acute medical hospitals. Clinicians may have difficulty translating theoretical knowledge of EDs into real life clinical situations, with management in medical wards seen as outside the scope of usual ward practice. This study investigated clinicians’ self-reported attitudes and knowledge about EDs to explore barriers to delivering care.

**Methods:** Clinicians at one large public hospital completed a 30-item online survey, based on the Theoretical Domains Framework, about general attitudes, knowledge and confidence when managing EDs.

**Results:** Of 166 clinicians who completed the survey across medical (n = 38), nursing (n = 64) and allied health disciplines (n = 62), 46% reported confidence in delivering care which dropped to 27% when the patient was perceived as “not motivated”. A limited number agreed/strongly agreed that they had the knowledge (42%), skills (27%), sufficient resources (18%) and training (14%) to deliver clinical care to people with EDs.

**Conclusion:** Consistent with previous research, clinicians did not feel skilled or knowledgeable when managing EDs in an acute hospital setting. Confidence to treat EDs is a barrier which is amplified if ED patients are perceived to resist the treatment on offer. Targeting identified barriers to improve knowledge and skills will improve the quality of care for EDs in an acute hospital setting. Education should focus on the ego-syntonic nature of the illness with awareness that resistance to treatment might be expected rather than an unusual behaviour during admission."

## O26. Developing a mental health training program for general practitioners using a spaced, microlearning approach: educating GPs about eating disorders—a pilot study

### Phillip Aouad^1^, Anna Janssen^3^, Sally Corry^1^, Karen Spielman^1^, Veronica Gonzalez-Arce^1,2^, Emma Bryant^1^, Rachel Simeone^1^, Tim Shaw^3^, and Sarah Maguire^1,2^

#### ^1^InsideOut Institute (Faculty of Medicine and Health), The University of Sydney, Sydney, Australia; ^2^Sydney Local Health District, NSW Health, Sydney, NSW, Australia; ^3^Research in Implementation Science and eHealth (RISe) Group (Faculty of Medicine and Health), The University of Sydney, Sydney, NSW, Australia

##### **Correspondence:** Phillip Aouad (phillip.aouad@sydney.edu.au)

*Journal of Eating Disorder* 2024, **12(1):** O26

**Background:** Eating disorders (EDs), specifically anorexia nervosa, are associated with one of the highest mortality rates compared to other psychiatric illnesses. EDs also have exceedingly low early detection and intervention rates. Protracted start-to-treatment likely reduces the chance of full remission. Early intervention is impossible without early detection—and in Australia general practice is still best placed to ensure this. Unfortunately, general practitioners (GP) are incredibly time-poor and often lack ED-specific training, which can be time intensive.

**Aims:** The current study aimed to develop, pilot, and evaluate an online microlearning approach (short, spaced learning) to develop eating disorder identification and medical management skills in general practitioners.

**Methods:** Twenty-three GP’s aged 25–59, recruited online, participated in the pilot microlearning program. The program consisted of a series of 10-vignettes, designed to be delivered over 6–10-weeks.

**Results:** Over-half (56.5%) of participants worked in small (< 10) group practices, with 65% having between 3 and 10 years practice experience. The majority (82.6%) felt the program was highly feasible and 87% felt the microlearning approach was effective for learning about EDs. A significant increase in knowledge (Wilcoxon Signed-Ranks Test; Z = − 2.179, p = 0.029) was also noted. Overall, qualitative feedback was also positive.

**Conclusions:** Preliminary findings demonstrate GP willingness to engage in learning to identify and manage EDs within their practice. This microlearning approach to GP training is novel and shows promise for helping increase skills within primary care. Future research should focus on larger samples, with novice GPs, more complex cases, and specific topics of need.

## O27. Ten-session cognitive behavioural therapy (CBT-T) is highly effective for eating disorders in a community eating disorders clinic

### Bronwyn C. Raykos^1^, David M. Erceg-Hurn^1^, Peter McEvoy^1,2^

#### ^1^Centre for Clinical Interventions; ^2^Curtin University

##### **Correspondence:** Bronwyn C. Raykos (bronwyn.raykos@health.wa.gov.au)

*Journal of Eating Disorder* 2024, **12(1):** O27

**Background:** Ten-session cognitive behavioural therapy (CBT-T; Waller et al. 2020) is an effective treatment for non-underweight eating disorders. However, most evaluations of CBT-T have been conducted with patients with bulimia nervosa and binge eating disorder and it is unclear if CBT-T is effective for Atypical Anorexia Nervosa (A-AN). Comparisons between CBT-T and longer CBT-ED are also lacking.

**Objective:** The present study examined the effectiveness of CBT-T in a specialist public health setting for patients with non-underweight eating disorders and compared these outcomes to a matched, historical sample who completed CBT-ED.

**Method:** Consecutive patients (N = 95, 16 + years) with a confirmed eating disorder diagnosis, including patients with A-AN (n = 39), completed measures of eating disorder and related symptoms at pre- and post-treatment, at weekly CBT-T sessions, and at 1- and 3-month follow up.

**Hypotheses:** We predicted that: (1) patients would achieve significant reductions in eating disorder and related symptoms in CBT-T that would be maintained at follow-up.

**Results:** Intent to treat analyses showed that patients achieved large and significant improvements in eating disorder symptoms, clinical impairment, and mood from pre- to post-treatment, that were maintained at 1-month and 3-month follow up (effect sizes ranged from 1.74 to 2.33). Effect sizes were at least as large as the historical control group. Outcomes were moderated by diagnosis, with patients with A-AN achieving significant benefit from CBT-T, albeit less than those with other diagnoses. CBT-T is an efficient and effective intervention for eating disorders.

## O28. Evaluating the effectiveness of evidence-based online foundational eating disorder training for general practitioners

### Emily Hardman^1^, Dr Angelique Ralph^1^, Dr Beth Shelton^1^, Louise Dougherty^1^

#### ^1^National Eating Disorders Collaboration, Crows Nest, NSW, Australia

##### **Correspondence:** Emily Hardman (info@nedc.com.au); Louise Dougherty (louise.dougherty@nedc.com.au)

*Journal of Eating Disorder* 2024, **12(1):** O28

**Background:** A skilled workforce is required to deliver safe, evidence-based care for people experiencing eating disorders. However, health professionals such as general practitioners (GPs) receive limited tertiary eating disorders training. To equip GPs with knowledge and confidence to provide care for people experiencing eating disorders, the National Eating Disorders Collaboration developed a freely accessible, four-hour, purpose-built, evidence-based online foundational training for GPs (‘GP Core Skills’), launched in June 2021.

**Aim:** To evaluate the effectiveness of GP Core Skills on GPs’ knowledge and confidence to provide care for people experiencing eating disorders.

**Methods:** Quantitative data on GPs’ knowledge and confidence to provide care for people experiencing eating disorders (measured on a Likert scale) was collected via pre- and post-training questionnaires. Descriptive statistics were used to determine if these measures had increased at course completion, based on data collected over the six-month period from June to November 2021.

**Results:** Between June and November 2021, 154 GPs completed the training. Post-training, 96% of participants rated their knowledge of best practice medical care for people experiencing eating disorders as ‘good’ or ‘excellent’ (compared to 12% pre-training), and 89% rated their confidence in identifying and responding to eating disorders as ‘confident’ or ‘very confident’ (compared to 12% pre-training).

**Conclusion:** Completion of GP Core Skills improved GPs’ knowledge and confidence to provide care for people experiencing eating disorders. The availability of effective and accessible eating disorders training supports the development of a skilled workforce and the delivery of safe, evidence-based care to people experiencing eating disorders.

## O29. Evaluating the feasibility of a brief, supported eTherapy for binge-eating disorder: a pilot study

### Sean Rom (BPsychSci (Hons)^1,2^, Jane Miskovic-Wheatley (DCP)^1^, Sarah Barakat (PhD Candidate)^1^, Sarah Maguire (PhD)^1^, Matthew Fuller-Tyszkiewicz (PhD)^2^

#### ^1^InsideOut Institute for Eating Disorders, Faculty of Medicine, Crows Nest, NSW, Australia; The University of Sydney, Sydney, NSW, Australia; ^2^School of Psychology, Faculty of Health, Deakin University, Geelong, VIC, Australia

##### **Correspondence:** Sean Rom (BPsychSci (Hons) (sean.rom@sydney.edu.au)

*Journal of Eating Disorder* 2024, **12(1):** O29

For individuals with binge eating disorder (BED), access to evidence-based, face-to-face treatment is often stymied by service-side (i.e., treatment availability and cost) and client-side barriers (i.e., self-stigma), contributing to low treatment uptake. Online self-help programs (eTherapy) address these barriers and have emerging support as effective treatment interventions; however, program evaluation in purely BED populations is limited, particularly with briefer programs that may have utility as early interventions. The aim of this study was to investigate the feasibility (i.e., program adherence and dropout) and potential efficacy of a brief, supported eTherapy in individuals with BED or subthreshold BED. Participants were 19 women with BED or subthreshold BED who completed a four-session eTherapy intervention employing the behavioural components of Cognitive Behavioural Therapy (CBT). Throughout this pre-post intervention study, participants completed weekly program content and questionnaires, self-monitored in a digital food diary, and attended weekly guided telehealth sessions with trained but non-expert guides. Program adherence was high compared to similar programs (92% of program content was completed) and statistically significant reductions were found across key outcome variables, as measured by the Eating Disorder Examination Questionnaire-Short (EDE-QS): objective binge episode days, loss of control over eating days, and eating disorder (ED) psychopathology, with an observed post-intervention average EDE-QS score below the cut-off indicative of ED. Results support the feasibility and potential efficacy of this brief eTherapy program in those with BED or subthreshold BED. Furthermore, findings suggest the program’s possible utility as an accessible and rapid acting early intervention or waitlist treatment.

## O30. Supporting families in proactive early intervention through eating disorders Victoria's carer coaching program

### Vicki Hams^1^, Julia Quin^1^, Helen Searle^1^, Susie Hansen^1^

#### ^1^Eating Disorders Victoria, Abbotsford, VIC, Australia

##### **Correspondence:** Vicki Hams (vlh235@gmail.com); Julia Quin (julia.quin@eatingdisorders.org.au); Helen Searle (helen.searle@eatingdisorders.org.au); Susie Hansen (susie.hansen@eatingdisorders.org.au)

*Journal of Eating Disorder* 2024, **12(1):** O30

In 2021, responding to the COVID-19 crisis of extensive waitlist to specialised eating disorder services in Victoria, Eating Disorders Victoria (EDV) was funded by Victoria’s Department of Health and Human Service (DHHS) to implement targeted approaches for supporting carers in early stages of illness of their loved one. The Carer Coaching Program employs carers with lived experience of supporting their own child through Family Based Treatment (FBT). Carer Coaching aims to support and guide parents through a structured psychoeducation and skills program to avoid deterioration in presentation while waiting for specialist intervention. The eight-week program empowers families to proactively respond to their loved one through support and practical skills that arm the family unit with skills to tackle critical early days of eating disorder treatment. As an adjunct, two skills-based group programs are being offered which is open to all.

In the past 12 months, 44 families have completed the program, with another 49 accessing one off support or a portion of the program. The two skills-based groups, RENEW and UPSKILL, have completed one round with 15 families.

Initial outcomes for the Eating Disorders Carer Coaching Program indicate families reporting a strong recovery. Of the 44 families 8 have had an admission subsequent admission to hospital. Early outcomes indicate a positive response to early psychoeducation and support, enabling families to proactively respond and at the very least hold their child from declining further while waiting for specialist interventions.

## O31. A systematic review of the relationship between orthorexia nervosa and obsessive-compulsive symptoms

### Phillipa Huynh^1^, Stephanie Miles^1^, Kathleen De Boer^1^, Maja Nedeljkovic^1^

#### ^1^Swinburne University of Technology, Hawthorn, VIC, Australia

##### **Correspondence:** Stephanie Miles (smiles@swin.edu.au)

*Journal of Eating Disorder* 2024, **12(1):** O31

Orthorexia nervosa (ON) is a proposed disorder driven by obsessive focus on “healthy” eating and compulsively following a rigid, restricted diet (Dunn and Bratman 2016). Recent theory and research on the orthorexia nervosa phenomenology have highlighted its similarities to both eating disorder and obsessive-compulsive disorder symptoms. However, existing research on ON has primarily been focused on eating disorder populations, and an understanding of the role of obsessive-compulsive symptoms is lacking. This systematic review aimed to analyse the literature on ON and obsessive-compulsive symptoms, critically evaluating the influence of populations studied and implemented ON assessments.

PsycINFO, PubMed and Web of Science databases were searched for peer reviewed research through to March 2022. Empirical studies published in English including measures for both ON and obsessive-compulsive symptoms were included. Of the 205 screened papers, 38 were included in the final review. It was found that research primarily sampled university student populations and/or health-focused individuals such as gym attendees and athletes with few studies investigating ON within clinical populations. Issues regarding the accurate assessment of ON were also noted. For instance, the most widely used ON assessment, the ORTO-15, lacks in items which target obsessive-compulsive symptoms (Donini et al. 2005). This systematic review highlights that the use of imperfect ON assessments in research has noteworthy implications for our understanding and treatment of ON.

## O32. New support group for carers/significant others of those experiencing severe & enduring eating disorders

### Helen Searle^1^, Vicki Hams^1^

#### ^1^Eating Disorders Victoria, Abbotsford, VIC, Australia

##### **Correspondence:** Helen Searle (helen.searle@eatingdisorders.org.au); Vicki Hams (vlh235@gmail.com)

*Journal of Eating Disorder* 2024, **12(1):** O32

In early 2022 Eating Disorders Victoria commenced the first Carer Support Group for Carers/Significant others of those experiencing severe and enduring eating disorders (SE-ED). Attendees were partners, family, friends or significant others of participants in the inaugural SE-ED 12-week recovery program, also run through EDV.

The Carer/Significant others course consisted of a 5-week structured program that was needs adapted to the participants. Both facilitators had lived experience of caring for someone with a SE-ED. It was anticipated that the carers and significant others of SE-ED participants would have different support needs to those of carers whose loved one is earlier on in the recovery journey. The course focused on individual agency and wellbeing for the Carers/Significant others through skills-based learning and mirrored some of the work that those undertaking the SE-ED recovery program were separately doing with EDV.

Weekly on-line surveys completed by the carers/significant others assessed whether their wellbeing was being impacted positively and whether they valued the content of the course overall.

Qualitative research regarding wellbeing and connection indicated that carers valued the opportunity to connect and meet in this way and valued the learning of new skills and shared experiences. Feedback was overwhelmingly positive such that participants requested an ongoing group to maintain connection, community and learning. An alum group will commence in May 2022.

## O33. Comparing treatment outcomes of outpatient group dialectical behaviour therapy for borderline personality disorder and anorexia nervosa

### Kathryn Althorpe^1,2^, and Dr Bethanie Gouldthorp^1,2^

#### ^1^Murdoch University, Perth, WA, Australia; ^2^Hollywood Private Hospital, Perth, WA, Australia

##### **Correspondence:** Kathryn Althorpe (Kathryn.Althorpe@health.wa.gov.au)

*Journal of Eating Disorder* 2024, **12(1):** O33

Anorexia nervosa (AN) is typically associated with maladaptive emotional over-control, yet patients commonly present with harmful behaviours typically associated with emotion dysregulation (e.g., self-harm). A recent shift in the conceptualisation of this as “emotional leakage” within an over-controlled personality type raises the clinical question of whether a standard Dialectical Behaviour Therapy (DBT) treatment approach that aims to target maladaptive emotional under-control, is indicated in addressing co-morbid symptoms of emotion dysregulation in patients with AN. It was hypothesised that patients with a diagnosis of AN would show lower pre-to-post treatment outcomes (emotion dysregulation, mood, severity of borderline symptoms) compared to patients with a presentation consistent with emotional under-control (i.e., borderline personality disorder [BPD]) following completion of a 12-week DBT group skills program. The sample included a total of 38 patients with an existing diagnosis of either AN or BPD. No significant differences between diagnostic categories were observed on any of the treatment outcome measures, with both groups showing significant improvements from pre-to-post in emotion dysregulation and mood, but not borderline symptomatology. These outcomes provide evidence that DBT Skills training is of benefit to patients with AN presenting with co-morbid self-harm, suicidality, or other symptoms of emotion dysregulation; and that treatment outcomes do not appear to be affected by potential differences in the formulation of these symptoms as part of an over-controlled (AN) versus under-controlled (BPD) personality type. This study contributes to the otherwise limited evidence available directing clinical decision making around treating co-morbid symptoms (e.g., self-harm) occurring for individuals with AN.

## O34. An Australian study evaluating the cost-effectiveness of the butterfly foundation residential eating disorders treatment (B-FREEDT) model of care

### Long Khanh-Dao Le^1^, PhD; Lidia Engel^2^, PhD; Mary Lou Chatterton^1^, PhD, Yong Yi Lee^1^, PhD, Phillipa Hay^2^, PhD, Deborah Mitchison^2^, PhD, Sinead Day^2^, Kathy Tannous^2^, PhD, and Cathrine Mihalopoulos^2^, PhD

#### ^1^Health Economics Division, School of Public Health and Preventive Medicine, Monash University, Australia; ^2^Western Sydney University, NSW, Australia

##### **Correspondence:** Long Khanh-Dao Le PhD (long.le@monash.edu)

*Journal of Eating Disorder* 2024, **12(1):** O34

The presentation will discuss details of protocol of economic evaluation project that attempts to: (i) establish the evidence base to support the case for a future funding mechanism to enable continued delivery of B-FREEDT in the Australian setting; (ii) assess whether B-FREEDT is cost-effective compared to treatment-as-usual (TAU) over short- to medium-term; and (iii) assess the extent to which the treatment program can be provided to those who cannot afford to pay for treatment themselves. The current economic evaluation methodology will comprise two stages: Stage 1: ‘Clinical trial-based economic evaluation’; and Stage 2: ‘Modelled evaluation’(see Fig. 1). The clinical trial-based economic evaluation will focus on data collected as part of the overall clinical trial’. It is anticipated that the benefits of the B-FREEDT may extend beyond the short time horizon of the trial-based evaluation. Therefore, an economic model will be developed to assess the longer-term costs and benefits of the B-FREEDT. Economic modeling is useful in circumstances where the longer-term impacts of intervention needs to be extrapolated beyond the short duration of trials/evaluations. Modeling can also be used to extrapolate the costs and benefits of interventions from the sample within a clinical trial to the broader population—this can include a comprehensive budget impact analysis. Together with economic protocol for the project, we also present overview of economic evidence for treatment of eating disorders including residential care treatment.

## O35. Prevalence of using weight loss supplement in children and adolescents: a systematic review

### Long Khanh-Dao Le^1^, PhD; Dhanushi Madhushani^2^, MPH; Natasha Hall^1^, MHE, and Cathrine Mihalopoulos^2^, PhD

#### ^1^Health Economics Division, School of Public Health and Preventive Medicine, Monash University, Australia; ^2^Deakin University, Geelong, Australia. Deakin Health Economics, Institute for Health Transformation, School of Health and Social Development, Australia

##### **Correspondence:** Long Khanh-Dao Le PhD (long.le@monash.edu)

*Journal of Eating Disorder* 2024, **12(1):** O35

**Background:** Many adolescents and young adults reported using over-the counter pills, herbal remedies, supplements, laxatives, and diuretics to aid dieting efforts. Many types of diet aids exist, including traditional diet pills or appetite suppressants, home remedies such as apple cider vinegar, and actively dangerous herbal supplements such as ephedra. Diet aid use is considered to be risky because the safety and efficacy of diet aids are unknown. The aim of this review to explore the prevalence of using diet aids in children, adolescents and young adults.

**Methods:** Electronic databases (including MEDLINE, CINALH, PsychInfo, and EMBASE) were searched for published studies reporting prevalence of diet pills, weight loss supplements and laxatives until December 2020. The Joanna Briggs Institute critical appraisal tool for prevalence studies was used to assess quality of included studies.

**Results:** One hundred and two articles were included. Most of studies were cross-sectional studies and conducted in high-income countries such as the United States, Canada and European countries. The majority of studies focus on general population and some studies focused on population with high risk for eating disorders. The prevalence of using weight loss supplements in children and adolescents ranges from 0 to 14.2%.

**Conclusions:** This study found that approximately one in ten children and adolescents has used diet aids. Preventive interventions for reducing the usage of these products is required.

## P1. Evaluating the effectiveness of group-based Radically Open Dialectical Behaviour Therapy as an adjunct to individual psychotherapy for the treatment of Anorexia Nervosa

### Bethanie Gouldthorp^1,2^, Shalane Sadri^1^, Bridget Visser^1^, Kathryn Althorpe^1,2^, and Stuart McCormack^1^

#### ^1^Hollywood Private Hospital, Nedlands, WA, Australia; ^2^Murdoch University, Murdoch, WA, Australia

##### **Correspondence:** Bethanie Gouldthorp (Bethanie.gouldthorp@health.wa.gov.au)

*Journal of Eating Disorder* 2024, **12(1):** P1

Anorexia nervosa (AN) is one of the most life-threatening mental disorders, with high relapse rates even with current best practice multi-disciplinary intervention. Further research into treatment for AN is critical to improve these outcomes. Radically-Open Dialectical Behaviour Therapy (RO-DBT) presents one approach through which treatment outcomes may be improved by addressing underlying maladaptive emotional over-control. This pilot project aimed to evaluate the feasibility and effectiveness of an adjunct outpatient RO-DBT skills group program for the treatment of patients with AN, alongside existing individual psychotherapy (treatment-as-usual (TAU)). It was anticipated that patients with AN who completed the group-based RO-DBT program as an adjunct to TAU would demonstrate improved treatment outcomes following completion of the program and at 9-week follow-up. The results of this study showed that patients in the RO-DBT group (N = 39) showed significant improvements post-treatment relative to pre-treatment, across a number of treatment outcomes including reduced severity of eating disorder symptoms, clinical perfectionism, and need for structure. These improvements were maintained at 9-week follow-up. These gains will be compared to existing evidence of standard treatment outcomes from individual CBT-E to evaluate the extent to which these treatment gains are over and above those expected for TAU. Drop-out rates and other factors associated with feasibility will also be explored. The initial data obtained in this study provides an important advancement in determining effectiveness of adjunct RO-DBT for treatment of maladaptive overcontrol in AN.

## P2. QuEDS: Innovations in measuring changes in eating behaviours and attitudes in an intensive eating disorder program

### Claire Gardiner^1^, Lucinda Morrow^1^, Morgan Sidari^1^ and Amy Hannigan^1^

#### ^1^Queensland Eating Disorder Service, Queensland Health, Herston, QLD, Australia

##### **Correspondence:** Claire Gardiner (claire.gardiner@health.qld.gov.au)

*Journal of Eating Disorder* 2024, **12(1):** P2

The QuEDS Day Program is a state-wide, non-residential, recovery focused intensive 8-week group program for people with eating disorders. Participants attend evidence based therapeutic groups and supported meal therapy, facilitated by a specialised multidisciplinary team. Various psychometric measures are used as part of standard clinical practice of the day program (EDE-Q, AAQ2, CIA, RAS-DS) however there was previously no way to measure change in eating behaviours.

In the absence of any validated tools, the Normal Eating Scale (NES) (Hart 2010) was deemed the most appropriate tool to use for the evaluation of change in eating behaviours and use was commenced as standard clinical practice in early 2021. The aim of the evaluation was to determine if there had been a change in specific eating attitudes/behaviours and/or if there was pattern/theme in the type of eating behaviours which shifted during the program.

The NES is a 43-question survey that asks participants to rate a statement on a 5-point Likert scale. The paper surveys were distributed prior to the day program starting and again at the end of the program. 29 participants over five-day programs commenced the day program between January 2021 and March 2022. A total of 18 clients completed the full day program and post NES data was missing for 1 client therefore only the data for the complete 17 clients was analysed for this report. To test for differences in normal eating behaviour pre- and post-program, we conducted a series of paired-sample t-tests and calculated effect sizes (Cohen’s d). The Holm-Bonferroni method was utilised to correct for multiple comparisons. All analyses were undertaken using R and R Studio.

Of the 43 items in the NES questionnaire, three items remained significant after correction for multiple comparisons. Namely, the day program significantly increased normal eating behaviours in the domains of importance of dietary fat intake (d = 0.87), reduced reading of nutrition labels (d = 0.83), and not restricting food to manage anxiety (d = 0.97). Moderate effect sizes were observed for an additional 16 items (in domains of money spent on food, increased flexibility in eating, decreased detailed focused eating habits and less impact on mood); however, these did not remain significant after correcting for multiple comparisons. The data from this project has provided insight and understanding around clients’ eating behaviours/patterns before and after the QuEDS day program.

## P3. Evaluating a solution-focused, skills-based intervention for carers of individuals with eating disorders

### Jessica Tone^1^, David Langford^1^, Belinda Chelius^1^

#### ^1^Eating Disorders Queensland

##### **Correspondence:** Jessica Tone (research@edq.org.au)

*Journal of Eating Disorder* 2024, **12(1):** P3

It is recognised that caring for a loved one with an eating disorder can have negative impacts on carer functioning and wellbeing, which in turn can lead to counterproductive caring behaviours and breakdown of interpersonal relationships. Extant research indicates that carer- or family-focused interventions can have positive effects on both caregiver wellbeing and patient outcomes. The integration of carer support services is therefore important when considering a service treatment model for eating disorders. Eating Disorders Queensland (EDQ) provides individual carer coaching to the key support people of individuals with eating disorders. Carer coaching sessions are solution focused and each session aims to collaboratively identify the key issues or challenges a carer is experiencing in supporting their loved one through eating disorder recovery and to provide the carer with practical skills and strategies. The aim of this program evaluation is to examine if this individual carer coaching service is effective in reducing carer depression, anxiety, stress, burnout, and isolation, and increasing carer confidence in providing support to their loved one. The impacts of the age of the carer’s loved one, their diagnosis, and the treatment approach employed in coaching sessions will be explored in relation to outcomes. Participants will understand the implementation of individual carer services within a service model for eating disorder recovery and the effectiveness of such approaches.

## P4. Low cholesterol as a risk factor for development of eating disorders

### Leanne Barron^1,2^, Robert Barron^4^, and Cameron Ward^3^

#### ^1^Queensland University of Technology Eating Disorders Clinic, Brisbane, QLD, Australia; ^2^The Banyans Medical Centre and Specialist Clinics, Bowen Hills, QLD, Australia; ^3^Queensland Children's Hospital, South Brisbane, QLD, Australia; ^4^Charles Sturt University, Bathurst, NSW, Australia

##### **Correspondence:** Leanne Barron (leanne.barron@qut.edu.au)

*Journal of Eating Disorder* 2024, **12(1):** P4

Cholesterol is a critical substance in the body, forming the root for synthesis of hormones, cortisol, bile salts, cell membranes and nerve sheaths. It is also a critical signalling molecule. Most people are able to manufacture cholesterol in vivo, but it will be proposed that many of those who develop eating disorders may be unable to synthesise adequate quantities. This could have significant implications for dietary choice and recommendations, given the low levels of cholesterol available particularly in vegan diets.

Interestingly, genetic studies have shown issues with cholesterol metabolism in patients with anorexia nervosa, and also in patients with autism spectrum disorder. Low cholesterol levels have been linked with depression, suicide and aggressive behaviour. It has been said that “Low cholesterol to a psychiatrist should be as significant as high cholesterol to a cardiologist”.

This presentation will discuss our previously published data showing the unusual bimodal distribution of cholesterol levels in patients with EDs, with 21% of values being above the reference range and 19% being below it. Monitoring random cholesterol levels as a tool in tracking recovery from the ED will be discussed, as will recommendations for an increased intake of cholesterol regardless of high or low levels.

Identification of elevated levels of 7 dehydrocholesterol (7DHC)—the immediate precursor of cholesterol—will be proposed, with implications in the potential aetiology of self-harm and behaviour issues.

## P5. Using a methylation model for nutritional motivation in patients with eating disorders

### Leanne Barron^1,2^, Robert Barron^4^, Cameron Ward^3^, Emily Gill^1^, and Esben Strodel^1^

#### ^1^Queensland University of Technology Eating Disorder Clinic, Brisbane, QLD, Australia; ^2^The Banyans Medical Centre and Specialist Clinics, Bown Hills, QLD, Australia; ^3^Queensland Children's Hospital, South Brisbane, QLD, Australia; ^4^Charles Sturt University (retired) Wagga Wagga, NSW, Australia

##### **Correspondence:** Leanne Barron (leanne.barron@qut.edu.au)

*Journal of Eating Disorder* 2024, **12(1):** P5

Eating disorders (EDs) are highly complex conditions with significant medical as well as psychological concerns. Patients who develop EDs are usually intelligent and motivated to be “healthy”, and thus a scientific approach can add to nutritional co-operation and motivation.

Methylation is an essential and highly nutrient dependent process, whereby gene expression is regulated, and synthesis of hormones and neurotransmitters occurs. Simple blood tests can be used to demonstrate issues in this process, and utilisation of a simple diagram can assist patients’ understanding of the role of nutritional co-factors in this process.

The MTHFR Single nucleotide Polymorphism (SNP) is inexpensive to measure and provides insight into a patient's innate methylation capacity. Variations in this SNP are common and require particular attention to specific nutrients.

Identification and explanation of individual nutritional susceptibilities and requirements, plus education regarding the benefits of specific nutrient containing foods, may assist patients in endeavouring to eat a greater variety of foods.

In practice, patients have been very interested in this process, and have been pleased to identify potentially underlying issues. They have been keen to incorporate this information into their overall treatment plan, discussing it with other members of their team.

## P6. Core needs: esteem, affiliation, and over evaluation of weight and shape

### Megan O’Shea^1,2^, Janette Brooks^1^, and Jason M. Sharbanee^1^

#### ^1^Discipline of Psychology, School of Arts and Humanities, Edith Cowan University, WA, Australia; ^2^BodyMatters Australasia, Cremorne, NSW, Australia

##### Corrspondence: Megan O’Shea (Megan@bodymatters.com.au)

*Journal of Eating Disorder* 2024, **12(1):** P6

Over-evaluation of weight and shape can have detrimental effects on physical and psychological health and can lead to maladaptive compensatory behaviours employed to create change in appearance. This study investigated possible predictors of negative over-evaluation of weight and shape. It was hypothesised that experiences of low esteem predict over-evaluation of weight and shape, as well as unmet affiliation needs predict over-evaluation of weight and shape. An exploratory hypothesis was that explicit and implicit measures of esteem and affiliation predict unique variance in over-evaluation of weight and shape. A total of 102 participants were involved, with 101 completing the two questionnaires, of these 36 participants also completed the Esteem Implicit Association Task (IAT), and 31 participants completing the Affiliation IAT which were explored with correlational analyses and multiple regression analyses. Results indicated unmet esteem needs have a strong, positive association with over-evaluation of weight and shape. A small-to-moderate, positive association was found between unmet affiliation needs and over-evaluation of weight and shape. Support was also given to the exploratory hypothesis that explicit and implicit measures predict unique variance. These results help underscore the role that unmet needs play in driving eating behaviour which could be an important determinant in eating disorder aetiology.

Keywords: weight and shape, esteem, affiliation, human needs.

## P7. Dimensional personality pathology and disordered eating in Australian young adults

### Tanya Gilmartin^1^, Caroline Gurvich^1^, Joanna F Dipnall^2,3^, and Gemma Sharp^1^

#### ^1^Monash Alfred Psychiatry Research Centre, Monash University and The Alfred Hospital, Melbourne, VIC, Australia; ^2^School of Public Health and Preventive Medicine, Monash University, Melbourne, VIC, Australia; ^3^Institute for Mental and Physical Health and Clinical Translation, School of Medicine, Deakin University, Geelong, VIC, Australia

##### Corespondence: Tanya Gilmartin (tanya.gilmartin1@monash.edu)

*Journal of Eating Disorder* 2024, **12(1):** P7

Eating disorders have been found to have a strong comorbidity with personality disorders. With the publication of the DSM-5, and alternative model for the diagnosis of personality disorders was developed, which conceptualised personality pathology as a dimensional, rather than categorical construct. The aim of this study was to explore how the DSM-5 alternate model traits predicted restrictive eating, binge-eating, purging, chewing and spitting, excessive exercising and muscle building among males and females. An online survey assessing disordered eating behaviours, PID-5 Short Form, and general psychopathology was completed by 394 females and 167 males aged between 16 and 30 years. Simultaneous equations path models were systematically generated for each disordered eating behaviour to identify how the PID-5 traits, body dissatisfaction and age predicted behaviour. The results revealed substantial differences between the statistical models developed for males and females. In addition, each disordered eating behaviour was found to be predicted by unique constellations of PID-5 subscales. In the context of past research, the results highlight the importance of considering the factors that may be associated with disordered eating behaviour when conducting an assessment of an individual with an eating disorder. Additionally, the current results provide support for a growing body of research that indicates differences between eating disorder presentations across the gender spectrum.

